# Requiescat in Pace

**Published:** 1899-12

**Authors:** 


### REQUIESCAT IN PACE.

AFTER a very short career, indeed, the new journalistic venture on the Pacific Coast has died of inanition. It promised much as a strong competitor of its Eastern rivals. It hoped for the affluence that has followed editorial direction, and it retires from the field with fresh honors of having lived the shortest life, made the fewest enemies, and sunk the least money of all its ill-timed predecessors. These are strange times; records are made in a day. Who will be the next?

